# Observer-Blind Randomized Crossover Trial of Yamamoto New Scalp Acupuncture in Diagnosis and Treatment Points for Neck Pain and Shoulder Stiffness in Healthy Participants

**DOI:** 10.1089/acu.2024.0003

**Published:** 2025-10-14

**Authors:** Soichiro Kaneko, Tetsuharu Kamiya, Shin Takayama, Ryutaro Arita, Rie Ono, Akiko Kikuchi, Minoru Ohsawa, Takuya Suematsu, Fumiya Omata, Sachie Kasukabe, Manami Suzuki, Tadashi Ishii

**Affiliations:** ^1^ Department of Kampo and Integrative Medicine, Tohoku University Graduate School of Medicine, Sendai, Japan.; ^2^ Department of Education and Support for Regional Medicine, Tohoku University Hospital, Sendai, Japan.; ^3^ Niigata University of Health and Welfare, Department of Acupuncture and Moxibustion, Niigata, Japan.; ^4^ Tohoku University School of Medicine, Sendai, Japan.

**Keywords:** Yamamoto New Scalp Acupuncture, Hoku diagnosis, stiff shoulders, pressure pain threshold

## Abstract

**Background:**

Yamamoto New Scalp Acupuncture (YNSA) uses microsystems in the body for various treatments. In YNSA diagnosis, Hoku is important for determining the treatment side. The A-point is used for treating neck and shoulder symptoms, and the D-point for low back pain; however, no studies support this.

**Aim:**

To investigate the site specificity of stimulation at the A-point and the usefulness of Hoku diagnosis.

**Methods:**

Nineteen healthy adults with no previous acupuncture experience were included. Participants randomly received three sessions of A-point, D-point, or no stimulation, followed by a 1-week minimum washout period. The visual analog scale (VAS) of shoulder stiffness, pressure pain threshold (PPT), and muscle hardness were evaluated at the bilateral shoulder region before, during, and 20 min after the stimulation, with the observer blinded to group allocation.

**Results:**

No significant differences were found in PPT between the three groups. Significant differences in VAS were observed between the three groups and in the comparison between the A-point and the control group. Intragroup analysis showed a significant improvement in the A-point and none in the D-point group. In subjects who had tenderness and induration on the right side of Hoku, the VAS score was significantly improved between the groups and multiple comparisons between the A-point and the control. The effect size was greater when limited by the Hoku diagnosis.

**Conclusion:**

The stimulation point selected according to the diagnosis point information might be more effective in YNSA. However, the results of this study were based on healthy adults, so the effect size was small. In the future, large-scale studies on patients with symptoms are needed.

## INTRODUCTION

Yamamoto New Scalp Acupuncture (YNSA) was originally developed as a microsystem acupuncture at the scalp. YNSA was developed by a Japanese doctor, Toshikatsu Yamamoto, and was first presented at a Japanese congress in 1973.^1–3^

The YNSA theory has nine treatment points (A–I) corresponding to body parts, known as basic points (Fig. 1A).^1–3^ There are basic points according to the symptoms in a specific body region. Physicians select treatment points depending on symptoms and body region. Although various approaches to acupuncture exist,^4^ YNSA is widely used in clinical practice for reasons ranging from refractory diseases to common symptoms because of the simplicity of the method from diagnosis to treatment and because traditional medical knowledge is not required.^1–3^

**FIG. 1.**
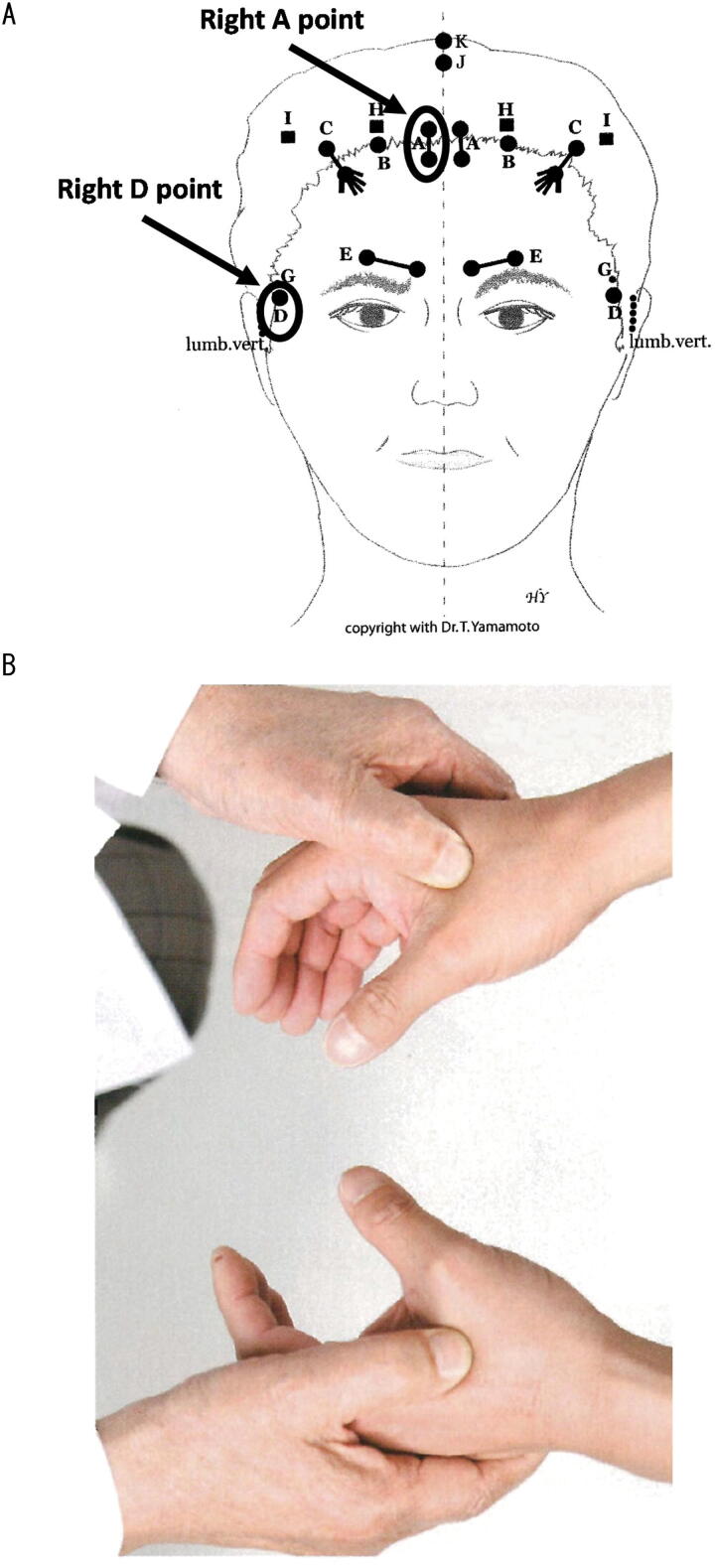
**(A)** Locations of stimulation points A and D.^2,3^ **(B)** Hoku diagnosis checking for Hoku points on bilateral sides.

As a first step in the clinical practice of YNSA, the treatment side is determined using the Hoku diagnosis. The practitioner palpates the Hoku (first dorsal interosseous muscle) on both sides, and the side with more induration/tenderness is selected for treatment (Fig. 1B). Second, basic points are selected as acupuncture points according to the location of the symptoms. For example, in case of a positive reaction at the right-Hoku point in a patient who complains of neck and shoulder stiffness, physicians select and stimulate the right basic A-point, which is used for the treatment of neck and shoulder symptoms.^2,3^

Evidence for YNSA treatment has not been well established. Only 13 articles were found in the PubMed database.^5–17^ Several clinical reports have shown some cases of effective use of YNSA for sudden sensorineural hearing loss, juvenile stroke, epilepsy, and Lyme disease.^6,9,13,16^ Several controlled trials on YNSA have reported neck and shoulder stiffness with related mood disturbance, chronic pain of degenerative osteoarthritis, migraine, acute non-specific low back pain, and stroke.^7,10–12,14,15^ However, these previous studies have not investigated the site specificity of the basic points of YNSA or the usefulness of Hoku diagnosis. In a previous study, we examined the safety of YNSA stimulation and the validity of the survey items. However, we were unable to investigate the site specificity and utility of Hoku diagnosis.^18^ Therefore, this study aimed to investigate the specificity of basic points and Hoku diagnosis.

## METHODS

### Design

This was an observer-blind randomized controlled crossover trial. In April 2014, the protocol was approved by the Ethics Committee of the Tohoku University Graduate School of Medicine (IRB No. 2014-1-009).

### Participants and Recruitment

Participants were recruited through advertisements from Tohoku University Hospital and Tohoku University School of Medicine from October 2014 to October 2015.

The participants were healthy volunteers aged between 20 and 70 years. A healthy volunteer was defined as a person who did not suffer from any specific chronic disease and had no difficulty performing daily activities. Participants provided written informed consent.

The inclusion criteria were as follows:

(1) May have stiff shoulders to the extent that it does not interfere with daily activities.
(2) Informed consent was provided by the patient.

The exclusion criteria are as follows:

(1) History of trauma to the head, neck, and upper limbs.
(2) History of chronic pain and disease requiring treatment.
(3) History of diabetes or systemic diseases.
(4) History of cardiovascular disease characterized by hypertension or hypotension.
(5) History of metal allergies
(6) Previous experience with acupuncture.
(7) Pregnancy.
(8) Receiving oral medication.

### Sample Size Calculations

This was a pilot study, and sample size calculations could not be performed because the effect sizes could not be calculated in the previous study. We recruited as many participants as possible from this setting.

### Randomization and Allocation

Sessions A (a session with acupuncture stimulation at point A, which is the stimulation point corresponding to the cervical-shoulder region), D (a session with acupuncture stimulation at point D, which is the stimulation point corresponding to the lower back region), and C (a control session without stimulation with the same time course) were scheduled. The participants were randomly allocated to sessions A, D, or C. Finally, participants attended all the sessions (A, D, and C), each in a crossover (Fig. 2). The washout period between sessions was at least 1 week. A random number table was used for the random assignment. The allocator concealed and controlled the order of the participant sessions from the evaluators.

**FIG. 2.**
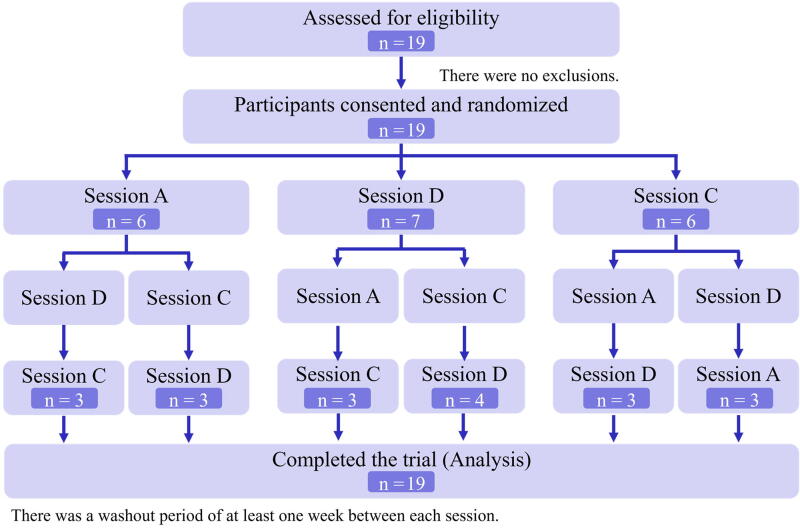
Flow diagram of this study. Participants are initially randomly assigned to session A, D, or C. They are then randomly assigned to a session they have not yet attended, and they complete all sessions A, D, and C.

### Blinding

The participants were unaware of their session assignments during the trial. Researchers, including statisticians, outcome assessors, and data analysts, were also blinded to the patients’ group assignments. The observer was blinded to needle location (Fig. 3). Although the acupuncturist was not blinded to the group assignments, it was not involved in outcome assessments or data analyses.

**FIG. 3.**
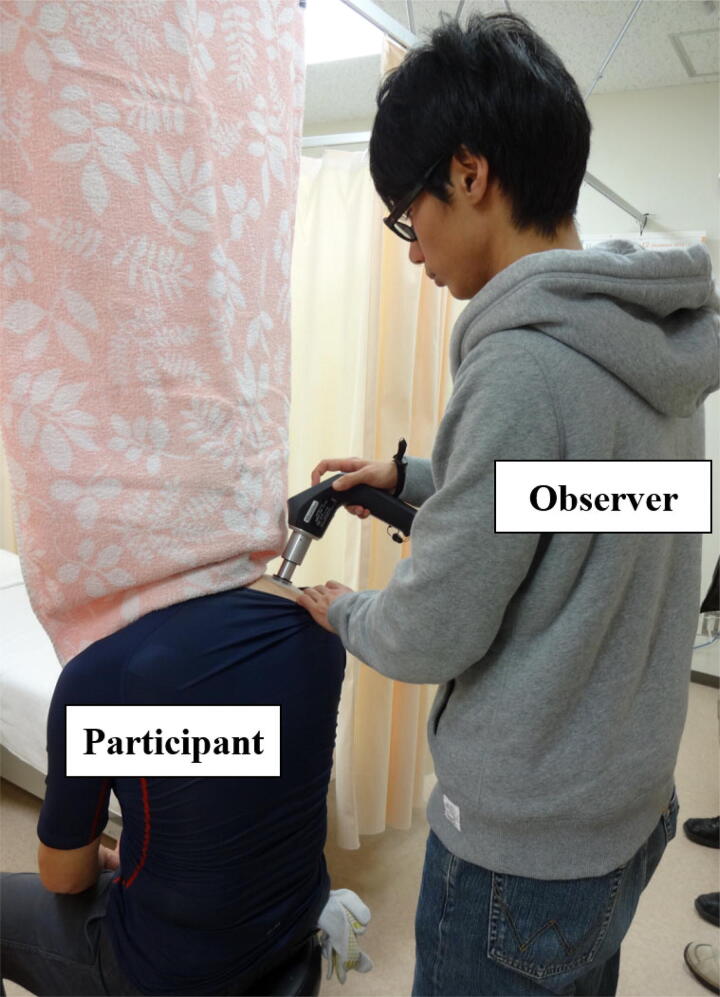
Pressure pain threshold (PPT) measurement. The stimulated area cannot be seen by the measurer.

### Intervention

The intervention was performed by a licensed acupuncturist who was a specialist in the YNSA treatment. The acupuncturist provided 20 min of stimulation on A-point or D-point on the right side using an acupuncture needle (0.25 mm in diameter and 40 mm in length; Serin Co.Ltd., Shizuoka, Japan). Acupuncture stimulation was not performed during the control session.

#### A-point

It is located approximately l cm bilateral to the midline, at the natural hairline, or approximately 5–6 cm frontal to the coronal suture. The A-points represent the head, cervical spine, and body parts supplied by nerves from this region. Indications for the YNSA basic A-point include relief of pain after an injury or postoperatively; headache and migraine of any origin; cervical syndrome; whiplash; vertigo; trigeminal or any facial, neck, or shoulder neuralgia; toothache before, during, or after dental treatment; stroke; and facial paralysis.^3^

#### D-point

The basic D-point is located in the temporal region, at the hairline approximately 1 cm above the zygomatic arch and 2 cm frontal to the ear, over the temporalis muscle. The D-point is bilateral. It represents the entire lower body and extremities. Indications for the YNSA basic D-point include lumbago, herniated intervertebral disc, circulatory disturbances of the legs, Raynaud’s syndrome, Parkinson’s syndrome, neuralgia rheumatism, arthritis, muscle cramps, sciatica, hemiplegia, paraplegia, multiple sclerosis, fractures, sprains, and gout.^3^

### Outcome Measures

The participants underwent the first measurement before the stimulation, after a 10-min rest, and the acupuncture needle was inserted later. During the 10-min needling, the participants underwent a second measurement. Finally, they underwent a third measurement after the withdrawal of the acupuncture needles 10 min later (Fig. 4).

**FIG. 4.**
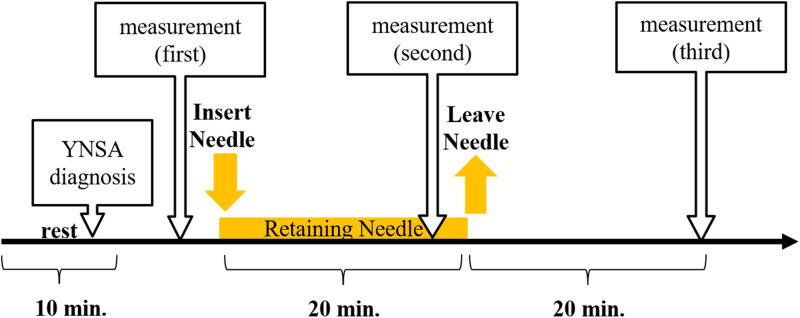
Time course of the experiment.

### Primary Outcomes

The primary outcome was pressure pain threshold (PPT; [kgf]) at the acupoint of Jianjing (GB21; Fig. 5).^19^ and the visual analog scale (VAS; [mm]) of subjective shoulder stiffness. Bilateral PPT was evaluated by a single observer using an ITO OE-220 system (Ito Physiotherapy & Rehabilitation; Kawaguchi, Saitama, Japan; Fig. 6A). Subjective shoulder stiffness was also measured in the left and right shoulders. The participants were kept in the same position with a unified eye view to reduce variations in the data due to changes in posture.

**FIG. 5.**
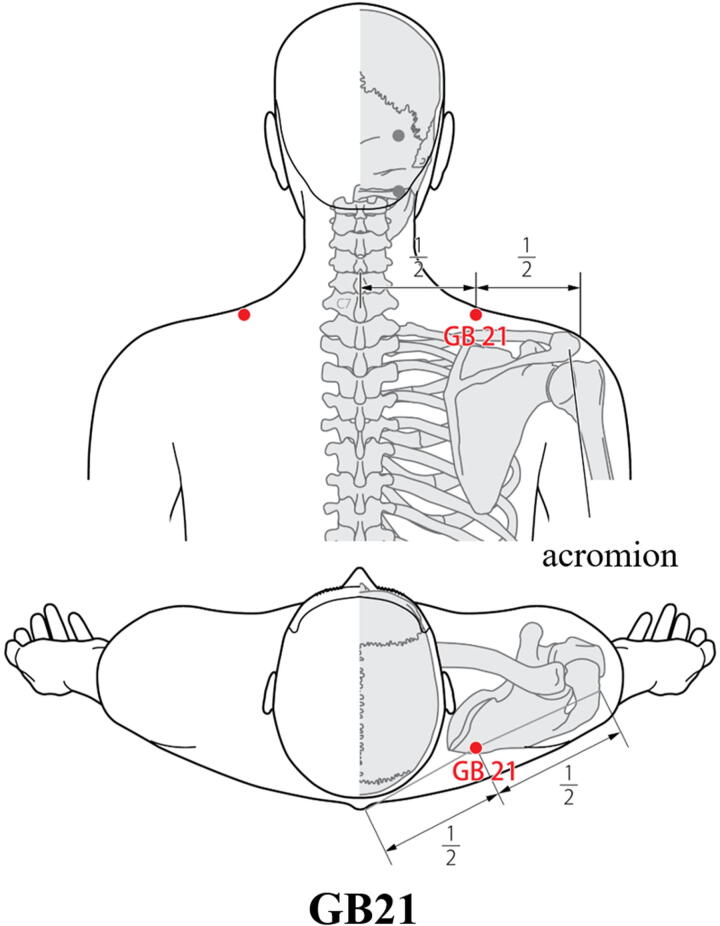
Location of the measurement point. Pressure pain threshold (PPT) and muscle hardness (MH) were measured at acupoint Jianjing (GB21). GB21 is located in the posterior region of the neck, at the midpoint of the line connecting the spinous process of the seventh cervical vertebra with the lateral end of the acromion.^19^

**FIG. 6.**
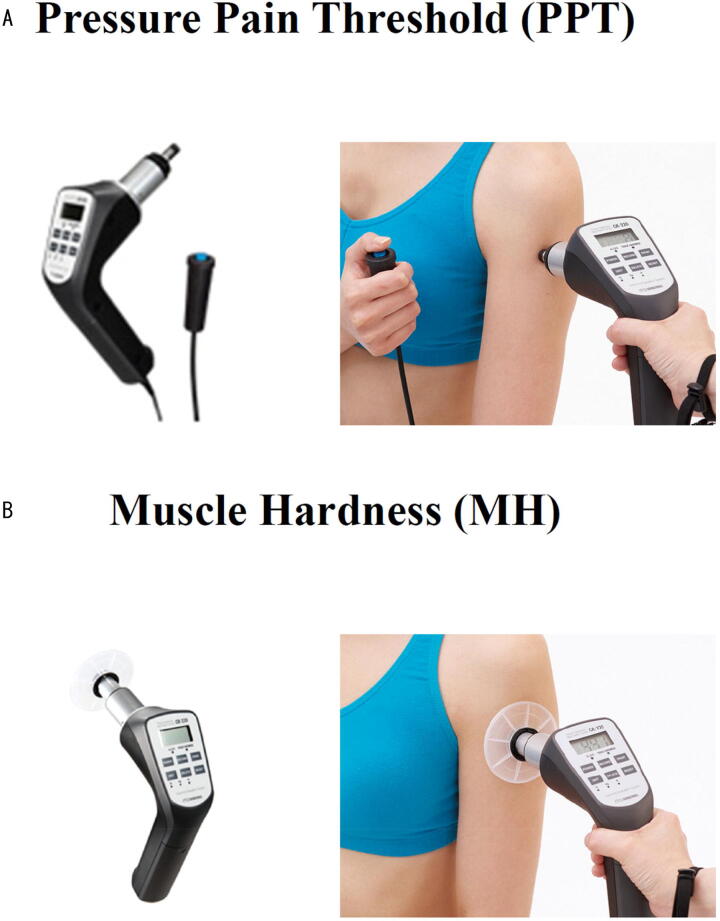
**(A)** Pressure pain threshold (PPT) and **(B)** muscle hardness (MH) measurement devices for shoulder stiffness. PPT and MH were evaluated using an ITO OE-220 system (Ito Physiotherapy & Rehabilitation, Kawaguchi, Saitama, Japan). PPT was measured by asking the participants to press a button when they felt pain and was recorded as the pressure when the pain was elicited by the device.

### Secondary Outcomes

Bilateral muscle hardness (MH; [kgf]) was also measured using the ITO OE-220 system. (Fig. 6B).

Systolic and diastolic blood pressure (SBP and DBP) and pulse rate (PR) were also measured at the same time.

### Hoku Diagnosis

Hoku diagnosis was performed by one acupuncturist at the beginning of each session (concurrent with the measurement of other parameters). Hoku diagnosis compares the stiffness/tenderness of the bilateral Hoku points (first dorsal interosseous muscle) using a technique to determine the stimulating side (Fig. 1B). The analysis is carried out when we do not consider the results of the Hoku diagnosis (all participants) and when we extract a person with a positive Hoku diagnosis (Hoku-positive participants).

### Statistical Analysis

For the analysis, we estimated the difference between the values measured before (first), during (second), and after the session (third). Two-way analysis of variance and multiple comparisons (adjusted by Bonferroni’s method), Kruskal–Wallis analysis, and multiple comparisons (Dunn test) were performed. Intergroup and intragroup comparisons used analysis of variance, where the effect size *η*^2^ was calculated by the sum of squares between groups and the sum of all squares.^20^ All statistical analyses were performed using IBM SPSS Statistics 27 software (IBM SPSS Statistics, Chicago, IL), with 95% statistical significance. Data are presented as the mean ± standard deviation (SD).

## RESULTS

In total, 19 participants (9 men and 10 women) with a mean age of 23.0 ± 3.8 years were enrolled in the study (Table 1). Eight participants (five men and three women) were diagnosed as right-Hoku positive. The background characteristics showed no significant differences in each group, except for DBP.

**Table 1.** Characteristics of Participants at Baseline

	Total (*n* = 19)	*p*-Value
Age	23.6 (5.7)	
Sex (male:female)	9:10	
Height (cm)	167.2 (7.5)	
Weight (kg)	59.5 (8.1)	
SBP (mmHg)		
A-point	113.0 (10.8)	0.158
D-point	115.4 (10.6)	
Control	112.1 (8.3)	
DBP (mmHg)		
A-point	71.4 (9.8)	0.048^*^
D-point	73.4 (9.9)	
Control	69.8 (7.4)	
PR (/min)		
A-point	71.0 (8.0)	0.167
D-point	72.7 (10.0)	
Control	69.3 (7.0)	
VAS (mm)		
A-point	15.5 (15.8)	0.960
D-point	14.8 (16.0)	
Control	14.9 (15.7)	
PPT (kgf)		
A-point	1.51 (0.56)	0.055
D-point	1.66 (0.45)	
Control	1.62 (0.55)	
MH (kgf)		
A-point	38.4 (7.23)	0.746
D-point	38.1 (6.51)	
Control	39.1 (6.73)	

* Significant difference (*p* < 0.05). Data are expressed as mean (SD).

DBP, diastolic blood pressure; MH, muscle hardness; PPT, pressure pain threshold; PR, pulse rate; SBP, systolic blood pressure; VAS, visual analog scale.

No significant differences were found in the PPT between the three groups: A-point, D-point, and control (*p* = 0.942; Table 2). In the intensity of subjective shoulder stiffness (VAS), a significant difference was found between the three groups (*p* = 0.033, 0.009; at second measurement and at third measurement) and in the comparison between the A-point and the control groups (*p* = 0.027 and *p* = 0.007; first vs. second and first vs. third, respectively); no significant difference was found between the D-point and the control group (*p* = 0.543, 0.206). Intragroup analysis showed a significant improvement in A-point (*p* = 0.001) and no significant improvement in D-point (*p* = 0.143).

**Table 2.** Change in Measurements for All Participants (*n* = 19)

	First	Second	Third	*p*-Value	
				Intergroup	Multiple comparisons
SBP (mmHg)					
A-point	113.0 (10.8)	108.5 (7.0)	110.7 (8.0)	0.781	—
D-point	115.4 (10.6)	114.9 (11.1)	112.2 (8.5)		
Control	112.1 (8.3)	110.0 (8.5)	10.85 (4.8)		
DBP (mmHg)					
A-point	71.4 (9.8)	69.7 (6.8)	71.6 (8.0)	0.991	—
D-point	73.4 (9.9)	72.3 (8.8)	73.3 (8.7)		
Control	69.8 (7.4)	69.0 (8.2)	68.8 (6.8)		
PR (/min)					
A-point	71.0 (8.0)	69.5 (7.6)	70.2 (7.5)	0.978	—
D-point	72.7 (10.0)	72.2 (9.2)	72.1 (8.4)		
Control	69.3 (7.0)	69.9 (7.9)	70.4 (7.5)		
VAS (mm)					
A-point	15.5 (15.8)	12.7 (14.4)*	12.2 (13.1)*	0.033** 0.009***	A vs. C; 0.027**, 0.007*** A vs. D; 0.609, 0.668 D vs. C; 0.543, 0.206
D-point	14.8 (16.0)	13.2 (13.8)	12.3 (14.1)		
Control	14.9 (15.7)	14.8 (15.3)	15.8 (15.8)		
PPT (kgf)					
A-point	1.51 (0.56)	1.57 (0.59)	1.61 (0.59)	0.942	—
D-point	1.66 (0.45)	1.70 (0.55)	1.71 (0.50)		
Control	1.62 (0.55)	1.67 (0.54)	1.70 (0.57)		
MH (kgf)					
A-point	38.4 (7.3)	37.8 (7.5)	36.1 (5.8)	0.818	—
D-point	38.1 (6.5)	37.5 (5.8)	37.4 (7.3)		
Control	39.1 (6.7)	38.0 (6.2)	35.9 (7.3)		

Data are presented as mean (SD).

* Friedman test was performed for intragroup comparisons, and a significant difference (*p* < 0.05) was found.

** There was a significant difference (*p* < 0.05) in the comparison of first vs. second.

*** There was a significant difference (*p* < 0.05) in the comparison of first vs. third.

DBP, diastolic blood pressure; MH, muscle hardness; PPT, pressure pain threshold; PR, pulse rate; SBP, systolic blood pressure; VAS, visual analog scale.

In subjects who had tenderness and induration on the right side of Hoku (*n* = 16; Table 3), no significant differences were found in PPT between the groups (*p* = 0.317). The VAS was significantly improved between the groups (*p* = 0.045, 0.006) and multiple comparisons between the A-point and the control (*p* = 0.039, 0.005), but not significantly different between the D-point and the control (*p* = 0.820, 0.084). In the calculation of effect sizes, participants who diagnosed positive Hoku (*η*^2^ = 0.168) showed more improvement compared with all the participants (*η*^2^ = 0.083).

**Table 3.** Change of VAS in Measurements Restricted to Hoku-Positive Participants (*n* = 8)

	First	Second	Third	*p*-Value	
				Intergroup	Multiple comparisons
VAS (mm)					
A-point	17.8 (16.8)	14.0 (15.9)*	13.8 (14.6)*	0.045** 0.006***	A vs. C; 0.039**, 0.005*** A vs. D; 0.494, 1.000 D vs. C; 0.820, 0.084
D-point	17.8 (18.3)	16.0 (15.3)	14.1 (14.3)		
Control	16.3 (17.9)	16.9 (17.7)	17.4 (17.6)		

Data are presented as mean (SD).

* Friedman test was performed for intragroup comparisons, and a significant difference (*p* < 0.05) was found.

** There was a significant difference (*p* < 0.05) in the comparison of first vs. second.

*** There was a significant difference (*p* < 0.05) in the comparison of first vs. third.

VAS, visual analog scale.

No significant differences were found for other parameters (SBP: *F*(4,162) = 0.439, *p* = 0.781; DBP: *F*(4,162) = 0.072, *p* = 0.991; PR: *F*(4,162) = 0.113, *p* = 0.978; MH: *F*(4,333) = 0.387, *p* = 0.818). No adverse effects including bleeding, infection, or pain were observed throughout the trial.

## DISCUSSION

In this study, we demonstrated the site specificity of the A-point and the usefulness of the Hoku diagnosis.

### Site Specificity

In the PPT and VAS analyses, neither the total analysis nor an additional analysis limited to Hoku-positive individuals found significant differences between the A- and D-point stimulations. However, in a comparison of subjective shoulder stiffness (VAS), A-point stimulation showed a significant improvement compared with the no-stimulation control; however, D-point stimulation did not. In addition, intragroup comparisons showed a significant decrease in VAS score for A-point stimulation, whereas no significant change was found for D-point stimulation. This result suggests that stimulation of the A-point is specific to shoulder stiffness.

### The Changes in PPT

The results show that there were no significant differences in PPT between the groups. While the lack of significant differences in PPT between the groups may suggest that acupuncture did not have a measurable effect on PPTs, it is important to consider the specific population involved in this study. Since this research was conducted on healthy adults, who do not exhibit the reduced pain thresholds typically seen in chronic pain patients, it is likely that significant changes in PPT were more difficult to detect. Unlike chronic pain patients, who often display central sensitization and a lowered pain threshold, healthy adults tend to have stable and higher baseline PPTs.^21,22^ Furthermore, while PPT is a commonly used measure for assessing mechanical pain sensitivity, acupuncture’s effects may extend beyond mechanical pain modulation, influencing neurophysiological pathways such as central sensitization or neuroinflammatory responses. These dimensions of pain perception are not always fully captured by PPT measurements. Future studies could benefit from integrating both subjective assessments and other objective markers, such as neuroimaging or biochemical analyses, to better evaluate acupuncture’s broader therapeutic effects on pain.

### The Usefulness of the Hoku Diagnosis

In YNSA practice, the laterality of stimulation is determined by Hoku diagnosis. The YNSA is performed based on this information; for example, A-point stimulation is applied to a person with a positive right Hoku reaction and shoulder pain or stiffness. In this study, the effect size was larger when restricted to Hoku-positive participants. These results demonstrate the usefulness of Hoku’s diagnosis. In this study, However, the magnitude of change in the degree of subjective shoulder stiffness is not large. Further research is needed to demonstrate the usefulness of the Hoku diagnosis.

The YNSA is highly appreciated worldwide; however, the complex relationship between the diagnosis reaction at each point and the treatment point has never been clarified. The results of our study are the first step toward clarifying the mechanism of YNSA.

### Mechanism of YNSA Stimulation

Studies on the mechanism of scalp acupuncture involving YNSA include one study by Shi et al., who demonstrated a mechanism whereby scalp acupuncture led to the activation of the Wnt/β-catenin signaling pathway, promoting angiogenetic factor expression and restoring blood perfusion in the ischemic zone.^23^ Zheng et al. reported the effects of scalp acupuncture on blood–brain barrier permeability, electroencephalogram and electroencephalographic potentials, blood biochemistry, and metabolism in an animal stroke model.^24^ Xu et al. reported that acupuncture stimulation of the head had an effect on depression in a rat model and that the protection of brain neurons was behind this effect.^25^ In other words, acupuncture stimulation of the scalp may affect the central nervous system in the same manner as acupuncture stimulation of the limbs and trunk. Further clarification of the mechanism of scalp acupuncture involving YNSA is needed.

### Benefits of Using YNSA Treatment for Shoulder Stiffness

YNSA treatment involved acupuncture stimulation of the scalp. Acupuncture was not applied locally to the central trapezius. The advantages of using YNSA treatment for shoulder stiffness may be due to the following: (1) there is no risk of pneumothorax, the most important adverse event of acupuncture, and (2) ability to assess the effects of treatment on pain during neck movement. The results of previous studies,^18^ together with the present one, indicate that the A-point in YNSA treatment is a safe and effective therapeutic approach for shoulder stiffness.

### Limitations

There are several limitations to this study, in particular the small sample size for the positive Hoku diagnosis points, and the fact that although statistically significant differences were observed, the changes in the degree of subjective symptoms were small from a clinical perspective. This is thought to be because the participants in this study were healthy and did not require treatment. In the future, it will be necessary to conduct clinical research targeting participants with symptoms.

## CONCLUSION

There is a site specificity of the YNSA stimulation points for subjective improvement. Hoku diagnosis also contributes to determining the appropriate stimulation side. For proper treatment, a combination of diagnosis and selection of the stimulation point might be considered in YNSA. However, the results of this study were based on healthy adults, so the effect size was small. In the future, large-scale studies on patients with symptoms are needed.
